# Meningococcal carriage in periods of high and low invasive meningococcal disease incidence in the UK: comparison of UKMenCar1–4 cross-sectional survey results

**DOI:** 10.1016/S1473-3099(20)30842-2

**Published:** 2021-05

**Authors:** Jenny M MacLennan, Charlene M C Rodrigues, Holly B Bratcher, Aiswarya Lekshmi, Adam Finn, Jenny Oliver, Mandy Wootton, Samantha Ray, Claire Cameron, Andrew Smith, Paul T Heath, Angela Bartolf, Tracey Nolan, Stephen Hughes, Anu Varghese, Matthew D Snape, Richard Sewell, Richard Cunningham, Alison Stolton, Carole Kay, Karen Palmer, David Baxter, Debbie Suggitt, Christos S Zipitis, Nicola Pemberton, Keith A Jolley, James E Bray, Odile B Harrison, Shamez N Ladhani, Andrew J Pollard, Raymond Borrow, Stephen J Gray, Caroline Trotter, Martin C J Maiden

**Affiliations:** a Department of Zoology, Peter Medawar Building for Pathogen Research, University of Oxford, Oxford, UK; b Meningococcal Reference Unit, Public Health England, Manchester Public Health Laboratory, Manchester Royal Infirmary, Manchester, UK; c School of Cellular and Molecular Medicine, University of Bristol, Bristol, UK; d Division of Public Health Wales, Temple of Peace and Health, Cardiff, UK; e NHS National Services Scotland, Health Protection Scotland, Glasgow, UK; f Glasgow Dental School, University of Glasgow, UK; g Scottish Microbiology Reference Laboratory, NHS Greater Glasgow & Clyde, Glasgow, UK; h St George's Vaccine Institute, Institute of Infection & Immunity, St George's University of London, London, UK; i Paediatric Infectious Diseases Research Group, St George's University of London, London, UK; j Research and Development Department, Maidstone and Tunbridge Wells NHS Trust, Maidstone, Kent, UK; k Central Manchester University Hospitals, NHS Foundation Trust, Manchester, UK; l Oxford Vaccine Group, Department of Paediatrics, University of Oxford and the National Institute for Health Research Oxford Biomedical Research Centre, Oxford, UK; m Microbiology Department, University Hospitals Plymouth NHS Trust, UK; n Lancashire and South Cumbria NHS Foundation Trust, Preston, Lancashire, UK; o Stockport NHS Foundation Trust, Stepping Hill Hospital, Stockport, UK; p Manchester Academic Health Science Centre, University of Manchester, Manchester, UK; q Department of Paediatrics, Wrightington Wigan and Leigh NHS Foundation Trust, Wigan, UK; r Clinical Trials Department, Wrightington Wigan and Leigh NHS Foundation Trust, Wigan, UK; s Immunisation and Countermeasures Division, Public Health England, London, UK; t Disease Dynamics Unit, Department of Veterinary Medicine, University of Cambridge, Cambridge, UK

## Abstract

**Background:**

The incidence of invasive meningococcal disease in the UK decreased by approximately four times from 1999 to 2014, with reductions in serogroup C and serogroup B disease. Lower serogroup C invasive meningococcal disease incidence was attributable to implementation of the meningococcal serogroup C conjugate vaccine in 1999, through direct and indirect protection, but no vaccine was implemented against serogroup B disease. UK Meningococcal Carriage surveys 1–3 (UKMenCar1–3), conducted in 1999, 2000, and 2001, were essential for understanding the impact of vaccination. To investigate the decline in invasive meningococcal disease incidence, we did a large oropharyngeal carriage survey in 2014–15, immediately before the changes to meningococcal vaccines in the UK national immunisation schedule.

**Methods:**

UKMenCar4 was a cross-sectional survey in adolescents aged 15–19 years who were enrolled from schools and colleges geographically local to one of 11 UK sampling centres between Sept 1, 2014, and March 30, 2015. Participants provided an oropharyngeal swab sample and completed a questionnaire on risk factors for carriage, including social behaviours. Samples were cultured for putative *Neisseria* spp, which were characterised with serogrouping and whole-genome sequencing. Data from this study were compared with the results from the UKMenCar1–3 surveys (1999–2001).

**Findings:**

From the 19 641 participants (11 332 female, 8242 male, 67 not stated) in UKMenCar4 with culturable swabs and completed risk-factor questionnaires, 1420 meningococci were isolated, with a carriage prevalence of 7·23% (95% CI 6·88–7·60). Carriage prevalence was substantially lower in UKMenCar4 than in the previous surveys: carriage prevalence was 16·6% (95% CI 15·89–17·22; 2306/13 901) in UKMenCar1 (1999), 17·6% (17·05–18·22; 2873/16 295) in UKMenCar2 (2000), and 18·7% (18·12–19·27; 3283/17 569) in UKMenCar3 (2001). Carriage prevalence was lower for all serogroups in UKMenCar4 than in UKMenCar1–3, except for serogroup Y, which was unchanged. The prevalence of carriage-promoting social behaviours decreased from 1999 to 2014–15, with individuals reporting regular cigarette smoking decreasing from 2932 (21·5%) of 13 650 to 2202 (11·2%) of 19 641, kissing in the past week from 6127 (44·8%) of 13 679 to 7320 (37·3%) of 19 641, and attendance at pubs and nightclubs in the past week from 8436 (62·1%) of 13 594 to 7662 (39·0%) of 19 641 (all p<0·0001).

**Interpretation:**

We show that meningococcal carriage prevalence in adolescents sampled nationally during a low incidence period (2014–15) was less than half of that in an equivalent population during a high incidence period (1999–2001). Disease and carriage caused by serogroup C was well controlled by ongoing vaccination. The prevalence of behaviours associated with carriage declined, suggesting that public health policies aimed at influencing behaviour might have further reduced disease.

**Funding:**

Wellcome Trust, UK Department of Health, and National Institute for Health Research.

## Introduction

Invasive meningococcal disease, caused by *Neisseria meningitidis,* is characterised by meningitis and sepsis worldwide, resulting in a rapidly developing, serious illness and death in otherwise healthy individuals.^1^ *N meningitidis* is a commensal organism frequently carried asymptomatically as part of a healthy oropharyngeal microbiota. Rates of carriage vary from 1–40% of the population depending on age and setting, peaking in adolescents and young adults in high-income countries.^2^ The relationship between meningococcal carriage and invasive meningococcal disease was systematically investigated in 1917 in military recruits by Captain James A Glover,^3^ who established that periods of overcrowding indoors were associated with increased meningococcal carriage, which in turn was associated with increased incidence of the disease. It has since been shown that some meningococci—for example, members of hyperinvasive lineages, including those defined as clonal complex 11, clonal complex 269, and clonal complex 41/44, and certain capsular groups (A, B, C, W, X, and Y)—are much more likely to cause disease than others and are carried at various rates in the global population.^4^, ^5^, ^6^, ^7^, ^8^ Therefore, it is essential to study carriage prevalence and invasive meningococcal disease incidence in the context of both serogroup and clonal complex.

**Research in context**

**Evidence before this study**

Many pharyngeal carriage studies have analysed meningococci circulating in the population, and have furthered understanding of meningococcal epidemiology and transmission. In high-income settings, carriage prevalence is highest in adolescents, who drive transmission in the wider population. In the UK, the success of the meningococcal serotype C conjugate (MCC) vaccination programme was, in large part, because of the reduction in serogroup C carriage in vaccinated individuals. This relationship was investigated by the UK Meningococcal Carriage (UKMenCar) 1–3 surveys, cross-sectional carriage studies in adolescents done before and after the MCC vaccine programme introduction in 1999. These surveys showed MCC vaccine-induced herd immunity against the epidemic strain (serogroup C, clonal complex 11) among adolescents. This herd immunity affected the transmission of clonal complex 11 meningococci and protected unvaccinated people. Independent of vaccine implementation, the relationship between invasive meningococcal disease risk and carriage prevalence is complex, varying with several factors, including strain types.

We did a literature review up to Feb 19, 2020, to identify studies that compared carriage rates between periods of high and low disease incidence in countries outside of Africa, where disease epidemiology is different to elsewhere. We searched PubMed with the Medical Subject Heading terms “meningococcal infections” AND “carrier state” AND “incidence” NOT “Africa”, which yielded 31 results; five studies were deemed relevant, which were in reasonably small populations (ie, not sampled nationally). Two studies reported a higher prevalence of carriage of the outbreak strain in high-incidence than in low-incidence areas, and three studies reported no differences. All languages of articles were included.

**Added value of this study**

The UKMenCar4 study was a large survey of meningococcal carriage among adolescents aged 15–19 years in the UK during a period of low national incidence of invasive meningococcal disease (2014–15). This study is similar to previous studies (UKMenCar1–3), which were done with the same methods but at a time of high disease incidence. Invasive meningococcal disease cases declined steadily between 1999 and 2014, including disease caused by serogroups not targeted by the MCC vaccine. We showed that adolescent carriage rates more than halved, from 16·7–18·7% in 1999–2001 to 7·2% in 2014–15. Risk factors for carriage were similar between UKMenCar1 and UKMenCar4, but adolescent participation in these risk factors declined, with fewer students in UKMenCar4 than in UKMenCar1 reporting active or passive smoking, intimate kissing, and attendance at pubs or nightclubs. This cross-sectional, observational study also reports baseline meningococcal carriage prevalence, stratified by serogroup and clonal complex, before the introduction into the UK national immunisation schedules of a conjugate polysaccharide vaccine against meningococcal serogroups A, C, W, and Y for adolescents, and protein-based capsular group B vaccines for infants. This long-term ecological study is, therefore, a reference point for the UK Be on the Team study (EudraCT number 2017–004609–42), a meningococcal carriage survey ongoing at the time of writing.

**Implications of all the available evidence**

This study illustrates the importance of understanding the epidemiology of meningococcal carriage in relation to disease and the potential for intervention. We showed the long-term effects of the MCC vaccine programme, with carriage rates of serogroup C meningococci among adolescents decreasing rapidly after MCC vaccine implementation and persisting at low levels 15 years later. Our observation that social behaviour and practices known to enhance meningococcal carriage have declined alongside carriage prevalence provides a plausible explanation for declining rates of the disease. Our results further suggest that public health policies aimed at influencing behaviour are likely to affect the incidence of invasive meningococcal disease. Assessing carriage prevalence is therefore an invaluable marker for assessing invasive meningococcal disease risk.

**Figure 1:**
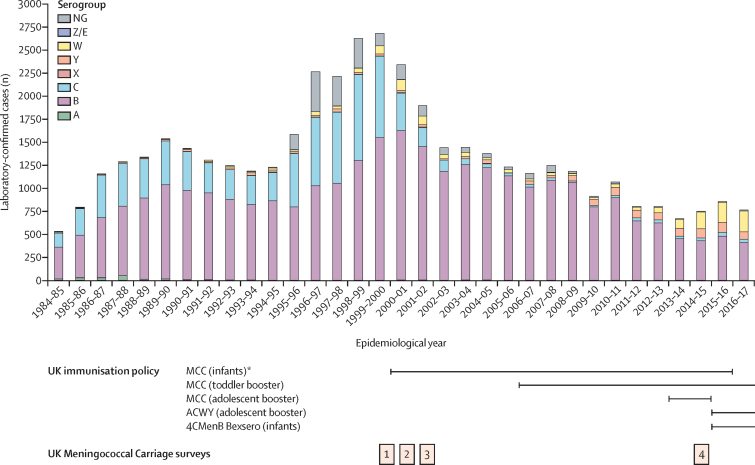
Invasive meningococcal disease incidence in the England and Wales from 1984–85 to 2016–17, by capsular serogroup Laboratory-confirmed disease cases, including culture-confirmed and PCR-confirmed disease, are stratified by epidemiological year. Coloured bars represent different capsular serogroups measured by dot-blot ELISA or *siaD* PCR. The lines below the graph show the changes in UK vaccination policy over this period for the MCC vaccine, the quadrivalent ACWY polysaccharide conjugate vaccine, and the protein-based vaccine 4CMenB (Bexsero [GlaxoSmithKline, Brentford, UK] is the only brand available). The timing of the four UK Meningococcal Carriage surveys are shown in relation to periods of high and low incidence of invasive meningococcal disease. Disease incidence data were obtained from the Meningococcal Reference Unit of Public Health England.^10^ Infant refers to those aged <12 months; toddler refers to those aged 12–13 months, and adolescent refers to those aged 13–15 years. *A catch-up campaign was implemented in 2000 to all individuals younger than 18 years, and in 2002 for all individuals younger than 25 years. 4CMenB=four-component meningococcal serogroup B. MCC=meningococcal serotype C conjugate.

Large-scale epidemics of meningococcal disease in the UK occurred during the two world wars and the Great Depression, with a small outbreak in the 1970s caused by a larger global pandemic of serogroup A invasive meningococcal disease.^9^ In the early 1980s, a sustained increase in invasive meningococcal disease incidence occurred, which peaked in the late 1990s for reasons that are incompletely understood (figure 1). Similar to many other high-income countries, serogroups B, C, W, and Y cause most invasive meningococcal disease in the UK.^1^ In 1999, meningococcal serogroup C conjugate (MCC) vaccines were introduced in response to an increase of serogroup C invasive meningococcal disease caused by clonal complex 11 meningococci (figure 1). The UK Meningococcal Carriage (UKMenCar) 1–3 surveys^8^, ^11^, ^12^ were done from 1999 to 2001 to measure the effect of the vaccination campaign on meningococcal carriage. These studies showed that the MCC vaccine was highly effective in reducing transmission of the epidemic meningococci.^8^, ^11^, ^12^ Furthermore, these investigations showed that transmission among adolescents (15–19 years), among whom the highest carriage prevalence was seen in the UK,^2^ was largely driven by social behaviours such as smoking, kissing, and attendance at pubs and nightclubs.^13^ The success of the UK MCC immunisation programme was largely attributable to herd protection, with significant and specific reduction in the carriage of meningococci from genogroup C and clonal complex 11.^8^, ^12^ In the years after implementation of the programme, however, there was also a steady decline in the yearly incidence of serogroup B invasive meningococcal disease, even though no vaccines against serogroup B were put in place for routine use in the UK.

The UK has a national health service (NHS) with a comprehensive public health disease surveillance system, allowing retrospective and prospective analysis of invasive meningococcal disease isolates. Since 2010, all disease-causing meningococcal isolates have been whole-genome sequenced by Public Health England (Manchester, UK) and the Scottish Microbiology Reference Laboratory (Glasgow, UK), with the sequences, deposited in the Meningococcal Genome Library.^9^ In combination with large collections of meningococcal carriage isolates, these sequence data provided an opportunity to compare carriage and disease-causing meningococci between periods of high and low incidence. To this end, a fourth national carriage survey (UKMenCar4) was done among adolescents in the UK to establish the prevalence and genetic diversity of carried meningococci in 2014–15, during which time there was a low incidence of invasive meningococcal disease. At this time, only the MCC vaccine was part of the national immunisation programme; in August, 2015, the MenACWY vaccine replaced the adolescent MCC booster dose for those approximately 14 years old, and in September, 2015, the four-component meningococcal serogroup B (4CMenB) vaccine was introduced into the national infant immunisation schedule (figure 1).^14^

## Methods

### Study design and participants

UKMenCar4 was a cross-sectional survey of meningococcal carriage in adolescents aged 15–19 years done from Sept 1, 2014, to March 30, 2015, using methods that were similar to those used for the three earlier surveys (UKMenCar1–3; figure 1).^8^, ^11^, ^12^, ^15^ In brief, UKMenCar1–3 were cross-sectional surveys of adolescents aged 15–19 years attending school or college, with sampling before (1999 [UKMenCar1]) or after (2000 [UKMenCar2] and 2001 [UKMenCar3]) the MCC vaccine was introduced across eight sampling regions^8^, ^12^ (appendix p 1). For the earlier surveys, an oropharyngeal swab was obtained from each participant for microbiological culture confirmation of meningococcal carriage,^8^, ^12^ and each participant completed a questionnaire to identify risk factors associated with carriage of *N meningitidis*^13^ (appendix pp 11, 12). For UKMenCar4, all adolescents aged 15–19 years who were attending school or college years 12 or 13, or equivalent, local to one of 11 sampling centres across the UK were eligible for inclusion (appendix p 1). Written informed consent was obtained from all study participants. Study participants were invited to provide an oropharyngeal swab for microbiological culture and whole-genome sequencing (WGS) analysis and to complete a risk factor questionnaire (appendix p 13).^15^ Questionnaires were excluded if essential data (identifier and age) were incomplete, or if valid consent was not obtained. The Office of National Statistics data was used to assess representativeness of the sampled population compared with the national population (appendix p 6). The study was approved under NHS Research Ethics Committee (reference number 14/SC/1163).

### Specimen collection, microbiological culture, and phenotypic characterisation

Laboratory protocols were replicated from the UKMenCar1–3 studies^8^, ^12^ to ensure direct comparability of the results of those surveys with the results of UKMenCar4.^15^ Students were recruited and the oropharynx of each participant was sampled with cotton or flocked swabs and cultured at each centre. The students then completed the questionnaire at school in clinics run by research staff. Swab samples were either plated directly onto selective media or placed in skim milk, tryptone, glucose, and glycerol (STGG) broth, and then all samples were sent to a microbiology laboratory at the local sampling centre for culture, storage, and later laboratory processing. If swabs placed into STGG broth could not be plated in the laboratory within 4 h they were frozen at −80°C, then thawed and cultured as soon as laboratory capacity allowed. After growth on selective media at 37°C and 5% CO_2_ for 16–24 h, a single colony with morphology typical of *Neisseria* spp was subcultured onto Columbia blood agar for a further 16–24 h at 37°C and 5% CO_2_. Candidate *Neisseria* spp were characterised by Gram staining and oxidase tests, and putative meningococci (Gram-negative, oxidase-positive diplococci) were stored at −80°C in brain heart infusion broth supplemented with glycerol.^15^ Other organisms were not systematically characterised. All Gram-negative, oxidase-positive diplococci were sent from the individual sampling centres to the Public Health England Meningococcal Reference Unit (Manchester, UK) for phenotypic (serological) characterisation of disease-associated serogroups (B, C, W, and Y).^16^

### WGS and genomic analysis

All Gram-negative, oxidase-positive diplococci underwent DNA extraction with the Qiagen DNeasy Blood & Tissue kit (Qiagen, Crawley, UK) at the Public Health England Meningococcal Reference Laboratory. High-quality, quantified chromosomal DNA underwent WGS at the Oxford Genomics Centre (Wellcome Trust Centre for Human Genetics, University of Oxford, Oxford, UK) using the HiSeq Illumina platform and validated protocols.

Short-read sequence data were assembled with Velvet version 1.2.10 or VelvetOptimiser software version 2.2.4^17^ (Department of Zoology, University of Oxford, Oxford, UK). The assembled high-quality draft genomes were uploaded onto PubMLST and annotated with *Neisseria* spp locus tag identifiers for more than 2400 individual loci using the automated BIGSdb software platform.^18^ Analysis with a gene-by-gene approach resulted in identification of *Neisseria* species by ribosomal multilocus sequence typing (rMLST),^19^ capsular genogroup (identified by capsule-specific alleles),^6^ and clonal complex by MLST.^20^ The participants' risk factor data were linked by participant identifier to their meningococcal genome on the PubMLST database isolate record. For UKMenCar1–3 surveys, genomic data were available from PCR amplification and direct nucleotide sequencing of MLST loci and capsular genes (*siaD*) and are available in the PubMLST database.^12^

### Statistical analysis

Previous multivariate analysis from the UKMenCar1–3 studies was used to identify risk factors and confounders (cigarette smoking; household exposure to cigarette smoke; intimate kissing; and socialising at pubs, clubs, and parties). For the power calculations, we estimated that a sample size of 18 000 participants was needed to provide 85% power to estimate the prevalence of rare strains. The sample size of the study was established by the ability to detect rare meningococcal strains (defined here as strains with a 1% prevalence). Assuming a 17% overall carriage prevalence (based on UKMenCar1–3), with 18 000 participants, we would expect to detect 3060 meningococci in total, and for rare strains at 1%, we would have a reasonably precise 95% CI of 0·85–1·15%. Data are presented for UKMenCar4 alongside the results from the UKMenCar1–3 surveys to enable comparison. Odds ratios (ORs), with two-sided 95% CIs, were calculated to compare carriage rates by age between 1999 (pre-MCC vaccine) and 2014–15. We estimated the point prevalence for each *Neisseria* spp, meningococcal serogroup, genogroup, and clonal complex with 95% CIs. Weighted proportions represent the carriage (%) by demographic stratum. Proportions were compared with χ^2^ or Fisher's exact tests. Prevalence ratios, with two-sided 95% CIs, were calculated to compare the populations of meningococci between 2014–15 and 1999 with respect to genogroup and proportions expressing capsules. Behavioural risk factors associated with meningococcal adolescent carriage were analysed with the use of univariate logistic regression. All analyses were done with R (version 3.5.2) and Stata (version 14).

### Role of the funding source

The funders of the study had no role in study design, data collection, data analysis, data interpretation, or writing of the report. The corresponding author had full access to all the data in the study and had final responsibility for the decision to submit for publication.

## Results

Between Sept 1, 2014, and March 30, 2015, 21 893 students were recruited across 11 UK centres. Oropharyngeal swabs were prepared for culture from 21 726 eligible students, of which 2085 participants' swabs were excluded as they were unable to be processed after shipping (n=467) or they were stored in incorrectly manufactured STGG broth (n=1618; appendix p 2). In total, 19 641 students' swabs and their corresponding risk-factor questionnaires were included in the analysis. The demographic characteristics of these students are summarised in the appendix (p 6). The proportion of the UK national birth cohort in school years 12 and 13 who were enrolled into UKMenCar4 was 1·74%; this sample was representative demographically of the entire population of that age in the UK in 2014–16 (appendix p 6).

Of 19 641 participants, 1420 had *N meningitidis* isolated, 246 had putative *Neisseria* spp isolated, and 17 975 had no *Neisseria* spp isolated (appendix p 2). Carriage prevalence of meningococci was 7·23% (95% CI 6·88–7·60; 1420/19 641) in UKMenCar4, 16·6% (95% CI 15·89–17·22; 2306/13 901) in UKMenCar1 (1999), 17·6% (17·05–18·22; 2873/16 295) in UKMenCar2 (2000), and 18·7% (18·12–19·27; 3283/17 569) in UKMenCar3 (2001). Rates for the individual centres ranged from 1·5% to 13·1% (appendix pp 3–4), with variation between centres similar to that observed in UKMenCar1–3. *Neisseria lactamica* was carried by 225 (1·15%, 95% CI 1·00–1·30) of 19 641 participants in UKMenCar4, compared with 132 (0·95% 0·80–1·12) of 13 901 in 1999. Other commensal *Neisseria* spp confirmed by ribosomal MLST included *Neisseria subflava* (15 isolates; carriage prevalence (0·08%, 95% CI 0·04–0·12), *Neisseria polysaccharea* (three isolates; 0·02%, 0·004–0·040), *Neisseria cinerea* (two isolates; 0·01%, 0·002–0·030), and *Neisseria bergeri* (one isolate, (0·005%, 0·0003–0·0300). 41 (0·21%) of 19 641 swabs with putative *Neisseria* spp could not be re-cultured for serological testing or DNA extraction for WGS, so they are not included in the putative *Neisseria* spp isolates.

**Figure 2:**
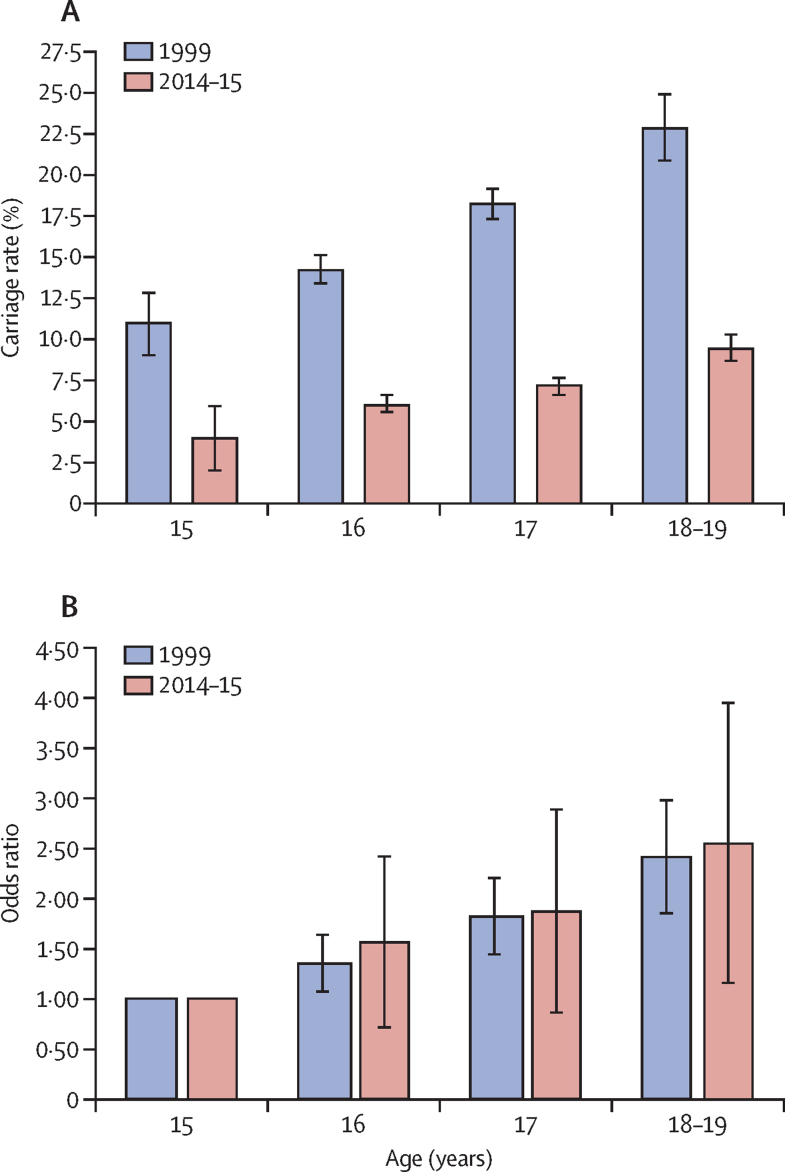
Carriage rates and odds ratios for carriage by age (A) Oropharyngeal carriage rates for adolescents aged 15–19 years in the UK for UKMenCar1 in 1999 (2306 meningococci isolates from 13 901 participants) and UKMenCar4 in 2014–15 (1420 meningococci isolates from 19 641 participants). The p values for the difference between the two surveys are p=0·00027 for the 15-year-old age group and p<0·0001 for all other age groups. (B) Odds ratios for carriage by age in the two carriage surveys; the data are also presented in appendix p 7. UKMenCar=UK Meningococcal Carriage.

Meningococcal carriage rate by age group showed an increasing trend from 15 years old to 18–19 years old, both in 1999 and in 2014–15 (figure 2A; appendix pp 6 and 8); however, the age-specific carriage rates were significantly lower in UKMenCar4 in every age group than in UKMenCar1 (1999), reflecting the overall decline in carriage (figure 2A). The age-specific ORs for carriage were similar for each age group between 1999 and 2014–15, and ORs increased with increasing age (figure 2B, appendix p 7).

**Figure 3:**
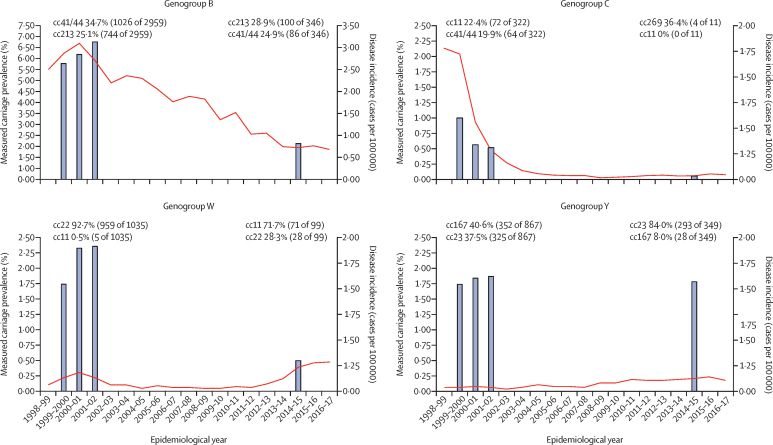
Association between adolescent meningococcal carriage prevalence and invasive disease incidence Bars indicate the measured carriage prevalence of meningococci in adolescents from UKMenCar1 (1999; 13 901 participants), UKMenCar2 (2000; 16 295 participants), UKMenCar3 (2001; 17 569 participants), and UKMenCar4 (2014–15; 19 641 participants; appendix p 9). Lines indicate England and Wales incidence (per 100 000 population) of invasive meningococcal disease caused by each genogroup over time. The predominant carried meningococcal clonal complexes in UKMenCar1–3 and UKMenCar4 are shown. The y-axes for genogroup B are scaled differently compared with those of the other genogroups to present the data more clearly. Disease incidence data were obtained from the Meningococcal Reference Unit of Public Health England.^10^ cc=clonal complex. UKMenCar=UK Meningococcal Carriage.

Capsular genogroup was identified from WGS data for 1370 (96%) of 1420 meningococci, of which all but one (<1%) were concordant with the phenotypic serogroup result, available in the PubMLST database.^12^ The measured UK carriage prevalence, from 19 641 eligible participants, by genogroup was 1·76% (95% CI 1·58–1·96; n=346) for genogroup B, 0·06% (0·03–0·10; n=11) for genogroup C, 0·50% (0·41–0·61; n=99) for genogroup W, 1·78% (1·60–1·97; n=349) for genogroup Y, 1·67% (1·50–1·86; n=328) for capsule null, and 0·90% (0·77–1·04; n=177) for genogroup E. Measured carriage prevalence for disease-causing genogroups decreased between 2001 and 2014–15 for genogroups B, C, and W, whereas genogroup Y prevalence did not change (figure 3; appendix p 9). The carriage prevalence of genogroup C reduced by 94%, from 1·00% (139/13 901) of meningococci in 1999 to 0·06% (11/19 641) in 2014–15.

The composition of carried meningococcal populations changed over time. As a proportion of the carried isolates identified, decreases were observed in genogroups B, C, and W between 2001 and 2014–15 (table). Increases were observed in the proportion of isolates that were genogroup Y and capsule null. Serogrouping (phenotypic) methods found that the proportion of meningococci in UKMenCar4 expressing capsule was 78·8% of genogroup W, 74·8% of genogroup Y, 55·8% of genogroup B, and 9·1% of genogroup C (table).

**Table:** Meningococcal carriage rates in adolescents aged 15–19 years in the UKMenCar1–4 surveys

		**UKMenCar1 (1999; n=13 901)**	**UKMenCar2 (2000; n=16 295)**	**UKMenCar3 (2001; n=17 569)**	**UKMenCar4 (2014–15; n=19 641)**	**Rate ratio 2014–15 *vs* 1999 (95% CI)**	**Rate ratio 2014–15 *vs* 2001 (95% CI)**	**p value for 2014–15 *vs* 2001**
Total meningococci		2306 (16·6%)	2873 (17·6%)	3283 (18·7%)	1420 (7·2%)	0·44 (0·41–0·46)	0·39 (0·36–0·41)	<0·0001
Serogroup B		..	..	..	..	..	..	..
	Genogroup	785/2306 (34·0%)	994/2873 (34·6%)	1180/3283 (35·9%)	346/1420 (24·4%)	0·72 (0·64–0·80)	0·68 (0·61–0·75)	<0·0001
	Capsule expression (% of total)	537/2306 (23·3%)	661/2873 (23·0%)	794/3283 (24·2%)	193/1420 (13·6%)	0·58 (0·50–0·68)	0·56 (0·49–0·65)	<0·0001
	Capsule expression (% of genogroup)	537/785 (68·4%)	661/994 (66·5)%	794/1180 (67·3%)	193/346 (55·8%)	..	..	..
Serogroup C		..	..	..	..	..	..	..
	Genogroup	139/2306 (6·0%)	92/2873 (3·2%)	91/3283 (2·8%)	11/1420 (0·8%)	0·13 (0·07–0·24)	0·26 (0·15–0·52)	<0·0001
	Capsule expression (% of total)	58/2306 (2·5%)	21/2873 (0·7%)	16/3283 (0·5%)	1/1420 (0·1%)	0·03 (0·00–0·20)	0·14 (0·02–1·09)	0·055
	Capsule expression (% of genogroup)	58/139 (41·7%)	21/92 (22·8%)	16/91 (17·6%)	1/11 (9·1%)	..	..	..
Serogroup W		..	..	..	..	..	..	..
	Genogroup	242/2306 (10·5%)	379/2873 (13·2%)	414/3283 (12·6%)	99/1420 (7·0%)	0·66 (0·53–0·83)	0·55 (0·45–0·68)	<0·0001
	Capsule expression (% of total)	146/2306 (6·3%)	217/2873 (7·6%)	236/3283 (7·2%)	78/1420 (5·5%)	0·87 (0·66–1·13)	0·76 (0·60–0·98)	0·052
	Capsule expression (% of genogroup)	146/242 (60·3%)	217/379 (57·3%)	236/414 (57·0%)	78/99 (78·8%)	..	..	..
Serogroup Y		..	..	..	..	..	..	..
	Genogroup	239/2306 (10·4%)	300/2873 (10·4%)	328/3283 (10·0%)	349/1420 (24·6%)	2·37 (2·04–2·76)	2·46 (2·14–2·82)	<0·0001
	Capsule expression (% of total)	131/2306 (5·7%)	163/2873 (5·7%)	184/3283 (5·6%)	261/1420 (18·4%)	3·24 (2·65–3·95)	3·28 (2·74–3·92)	<0·0001
	Capsule expression (% of genogroup)	131/239 (54·8%)	163/300 (54·3%)	184/328 (56·1%)	261/349 (74·8%)	..	..	..
Capsule null		..	..	..	..	..	..	..
	Genogroup	369/2306 (16·0%)	534/2873 (18·6%)	622/3283 (18·9%)	328/1420 (23·1%)	1·44 (1·26–1·65)	1·22 (1·08–1·37)	0·009
Other (including serogroups E, H, L, W/Y, X, and Z)		..	..	..	..	..	..	..
	Genogroup	532/2306 (23·1%)	574/2873 (20·0%)	643/3283 (19·7%)	287/1420 (20·2%)	0·87 (0·77–1·00)	1·03 (0·91–1·17)	0·715

Data are n/N (%), unless otherwise indicated. Serogroup was established for capsular groups B, C, W, and Y only. Other genogroups included: E, n=177; H, n=1; L, n=3; W/Y, n=4; X, n=20; and Z, n=32 in UKMenCar4. For UKMenCar1–3, the rates of other individual genogroups were not established. UKMenCar=UK Meningococcal Carriage.

**Figure 4:**
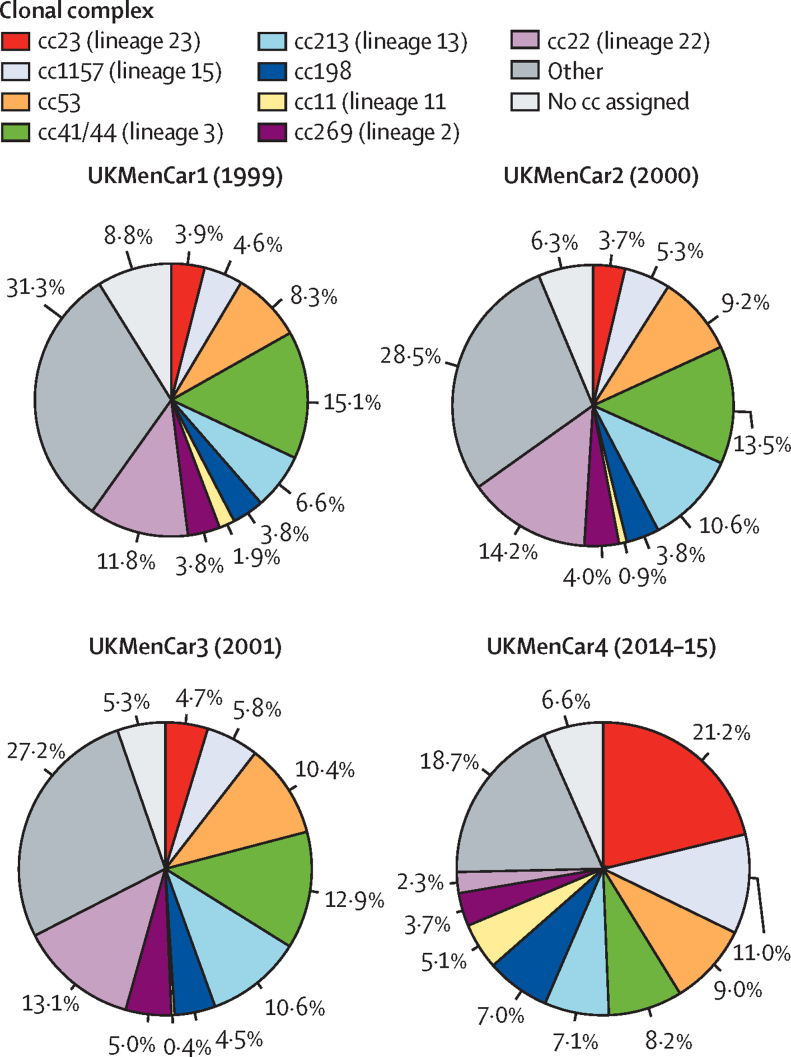
Distribution of meningococcal clonal complexes in UKMenCar1–4 surveys Proportions of the nine most frequently occurring clonal complexes of meningococci are shown for the four UKMenCar studies. A clonal complex was assigned to 2102 of 2306 meningococci in UKMenCar1, 2692 of 2873 in UKMenCar2, 3109 of 3283 in UKMenCar3, and 1326 of 1420 in UKMenCar4. cc=clonal complex. UKMenCar=UK Meningococcal Carriage.

Meningococcal isolates belonged to 29 clonal complexes, with clonal complexes for 94 (7%) of 1420 isolates not categorised because of incomplete MLST profiles or belonging to sequence types not yet assigned to clonal complexes (appendix p 5). Nine clonal complexes accounted for 1066 (75%) of 1420 isolates (figure 4), with measured carriage prevalence in 19 641 samples of 1·53% (95% CI 1·37–1·71; n=301) for clonal complex 23, 0·79% (0·68–0·92; n=156) for clonal complex 1157, 0·65% (0·55–0·77; n=128) for clonal complex 53, 0·59% (0·49–0·71; n=116) for clonal complex 41/44, 0·51% (0·42–0·62; n=101) for clonal complex 213, 0·51% (0·42–0·62; n=100) for clonal complex 198, 0·37% (0·29–0·47; n=73) for clonal complex 11, 0·26% (0·20–0·34; n=52) for clonal complex 269, and 0·17% (0·12–0·24; n=33) for clonal complex 22. The relative carriage prevalence of certain clonal complexes changed over time: clonal complexes 23, 1157, 198, and 11 increased from UKMenCar1 to UKMenCar4 (all p<0·001; appendix p 10; figure 4). Of the hyperinvasive lineages, clonal complex 23 had a relative carriage prevalence ratio (2014–15 to 1999) of 5·37 (95% CI 4·29–6·73) and clonal complex 11 had a ratio of 2·76 (1·90–4·00). Significant reductions were observed in the relative carriage prevalence (2014–15 to 1999) of clonal complexes 41/44 (rate ratio 0·54, 95% CI 0·44–0·66) and 22 (0·20, 0·14–0·28) meningococci from UKMenCar1 to UKMenCar4.

Invasive meningococcal disease incidence measured across all age groups, changed from 1999 to 2014–15 for each disease-associated serogroup, coincident with fluctuations in clonal complex distribution (figure 4) and adolescent carriage of these clonal complexes (figure 3). Serogroup B invasive meningococcal disease steadily declined from 1999 to 2014–15, with a shift in the predominant clonal complex carried by adolescents from clonal complex 41/44 to clonal complex 213 (figure 4). Rates of group C invasive meningococcal disease declined rapidly after MCC introduction in 1999, coincident with a decrease in genogroup C carriage and elimination from carriage of genogroup C clonal complex 11 by 2014–15 (figure 3). For genogroup W, the predominant clonal complex carried by adolescents switched from clonal complex 22 in UKMenCar1–3 to clonal complex 11 in UKMenCar4 (figure 3), which was coincident with increasing serogroup W disease (figure 1 and 3). For genogroup Y, the predominant clonal complex in UKMenCar4 was clonal complex 23, whereas clonal complex 167 predominated in 1999 (figure 3).

**Figure 5:**
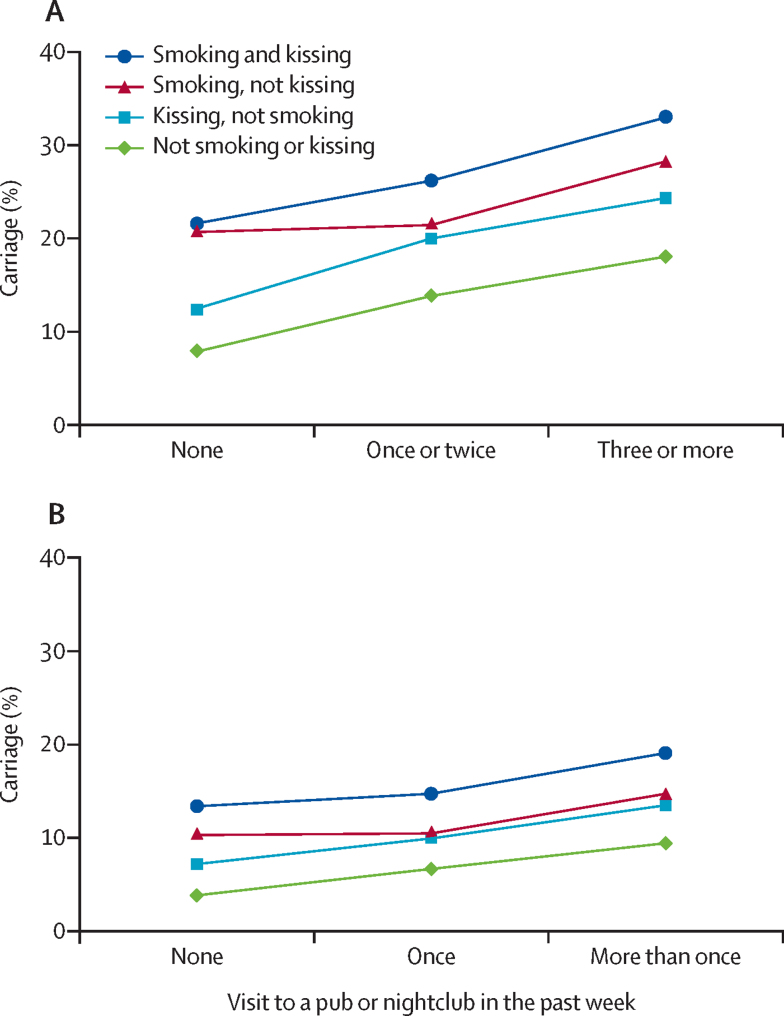
Risk factors for carriage among UK adolescents in UKMenCar1–4 (A) Meningococcal carriage rates by frequency of risk factors in UKMenCar1, the adolescent UK carriage survey done in 1999 (n=13 919). Figure reproduced unchanged under CC BY.^13^ (B) Meningococcal carriage rates by frequency of risk factors in UKMenCar4, the adolescent UK carriage survey done in 2014–15 (n=19 641). Visits to pubs or nightclubs were categorised differently between UKMenCar1 and UKMenCar4. UKMenCar=UK Meningococcal Carriage.

The proportion of adolescents self-reporting as regular smokers decreased by 47·9% from 1999 (21·5%; 2932/13 650) to 2014–15 (11·2%; 2202/19 641; p<0·0001), although the small proportion of heavy smokers (>20 cigarettes per day) was unchanged (0·3%, 46/13 650 in 1999 *vs* 0·3%, 55/19 641 in 2014–15; p=0·41). Reporting of intimate kissing in the previous week reduced by 16·7% from 1999 (44·8%; 6127/13 679) to 2014–15 (37·3%; 7320/19 641; p<0·0001), with fewer adolescents reporting multiple (more than one) partners in 2014–15 (4·6%; 904/19 641) than from 1999 (18·9%; 122/13 679; p<0·0001). Attendance at pubs and nightclubs fell by 37·2% in 1999 (62·1%; 8436/13 594) to 2014–15 (39·0%; 7662/19 641; p<0·0001). A cumulative pattern of risk factors was associated with higher carriage (figure 5), with adolescents who reported smoking, kissing, and visiting pubs or nightclubs more frequently having up to a five times higher risk of carriage than adolescents who did not participate in those activities. Similar to the odds ratios for carriage by age (figure 2B), the behavioural risk factors did not change; rather the prevalence of these behaviours changed (figure 5).

## Discussion

UKMenCar1–4 enabled the investigation of meningococcal epidemiology in the UK, providing national, cross-sectional estimates of carriage prevalence in adolescents, enabling the observation of changes over time of genotype replacement, and to facilitate the assessment of the effects of meningococcal vaccine programmes. UKMenCar4 additionally provided definitive molecular characterisation of meningococci carried by UK adolescents in 2014–15.

From 1999 to 2015, the incidence of invasive meningococcal disease in the UK declined, from 1856 laboratory-confirmed cases in England in 2001–02 to 724 in 2014–15, a 61% reduction.^21^ Older adolescents generally have the highest rates of meningococcal carriage among countries with similar epidemiology to western Europe.^2^ The UKMenCar4 survey identified a 61% reduction in measured carriage prevalence among UK adolescents since 2001, providing a potential explanation for the decrease in invasive meningococcal disease, because of the disruption of meningococcal transmission.^22^

These data are consistent with the long-term effectiveness of the MCC national immunisation programme. During the UKMenCar1–3 surveys, a significant reduction in carriage of genogroup and serogroup C meningococci was attributed to effects of the MCC vaccine.^8^, ^11^, ^12^, ^13^ In the UKMenCar4 survey, genogroup C meningococci occurred at a low frequency and genogroup C, clonal complex 11 meningococci were not identified, probably because of the combined effects of the MCC vaccine and reduction in carriage-promoting behaviours among adolescents. However, a degree of transmission persisted in the wider community as 28 cases of serogroup C invasive meningococcal disease were reported to Public Health England in 2014–15.^21^ Data from the Meningococcal Genome Library^9^ showed that of the 23 serogroup C isolates with sequencing data, 16 (70%) were clonal complex 11. This finding shows the importance of ongoing, real-time surveillance using national collections of invasive meningococcal disease genomes for assessing vaccination programmes.

With respect to hyperinvasive lineages, the relative carriage prevalence of genogroup C, clonal complex 11 in UKMenCar1 (1999) was low (1·5%),^12^ despite high rates of disease caused by this genotype. These data contrast with the relative carriage prevalence of 5·0% for genogroup W, clonal complex 11 in UKMenCar4 (2014–15), a strain that was associated with a national increase in serogroup W invasive meningococcal disease from 2009.^23^ Furthermore, 79% of genogroup W meningococci isolated in 2014–15 expressed a capsule, a proportion that was higher than those for genogroups B, C, and Y meningococci in 2014–15, but similar to that of genogroup C, clonal complex 11 in 1999 (81%),^12^ a factor probably contributing to the invasive potential of these meningococci. The rapid clonal expansion of genogroup W, clonal complex 11 was coincident with reductions in the carriage of genogroup W, clonal complex 22, which has a low disease potential.^4^ Increasing circulation of genogroup W, clonal complex 11 meningococci had a substantial effect on the incidence of serogroup W invasive meningococcal disease globally,^23^, ^24^ and subsequent^25^ vaccine policies in Australia (implemented in February, 2019), the Netherlands (implemented in October, 2018), and the UK (implemented in August, 2015) have largely targeted adolescents because they are the highest carriers of meningococci,^14^, ^24^, ^26^ and we have found high rates of carriage of genogroup W, clonal complex 11 in them. This study reinforces that the decision to vaccinate adolescents to protect the wider population against genogroup W, clonal complex 11 disease was justified.

A steady decrease in the total number of cases of serogroup B invasive meningococcal disease in England—from 1495 cases in 1999–2000 to 418 cases in 2014–15 (a 72·0% reduction)—occurred in the absence of an effective vaccination programme targeting serogroup B meningococci in the UK. By comparison, there was a 96·7% reduction in cases of serogroup C invasive meningococcal disease over the same period following MCC vaccine introduction.^21^ We observed a 69% reduction in genogroup B carriage prevalence among adolescents between UKMenCar1 and UKMenCar4; this reduction probably contributed to reductions in serogroup B invasive meningococcal disease in both children younger than 5 years and adolescents, both groups at a high risk of the disease.^22^ Additional factors for the declining incidence of serogroup B invasive meningococcal disease include the reduction in the carriage of hyperinvasive clonal complex 41/44, with relative increases in clonal complexes with lower invasive potential,^4^ and a lower proportion of meningococci expressing capsule. In 2019, the global burden of serogroup B invasive meningococcal disease was low,^5^ with evidence of declining incidence rates worldwide^27^ often in the absence of vaccination programmes. This decrease implicates secular changes in the circulation of hyperinvasive serogroup B meningococci, as shown in the UK (figure 3), as a reason for reduced disease rates (figure 1). The protein-based 4CMenB vaccine Bexsero was introduced into the infant immunisation programmes in Andorra, Ireland, Italy, Lithuania, and the UK to provide direct protection to this susceptible group.^24^ There was no evidence of herd immunity induced by the 4CMenB vaccine to disease-causing serogroups (B, C, W, or Y) in an Australian intervention study,^28^ and at the time of writing, a large-scale UK carriage study was further investigating herd immunity for both licenced protein-based vaccines (EudraCT number 2017-004609-42). In the absence of any evidence of herd effects, it might not be cost-effective to immunise adolescents. Therefore, identifying other less costly interventions might be necessary.

Increasing incidence of serogroup Y invasive meningococcal disease was reported to Public Health England from 2011–12 to 2019, particularly affecting the older (aged 65 years or older) population. In UKMenCar4, genogroup Y, clonal complex 23 meningococci had replaced the less invasive genogroup Y, clonal complex 167 meningococci and genogroup Y, clonal complex 22 meningococci that were present in UKMenCar1–3. Indirect protective effects of conjugate polysaccharide vaccines were observed with the MCC vaccine globally and with the serogroup A vaccine in the African meningitis belt (MenAfriVac).^29^ The quadrivalent, conjugate polysaccharide MenACWY vaccine might prevent the acquisition of meningococci,^30^ thereby reducing onwards transmission, which could reduce the increasing rates of serogroup Y invasive meningococcal disease in the UK via inclusion of the vaccine in the adolescent programme introduced in 2015.^14^

We observed changes in adolescent behaviour between UKMenCar1 and UKMenCar4, although the incremental increase in risk of carriage with increasing age and age-associated odds ratios for carriage were similar between 1999 and 2014–15. The decline in adolescent carriage prevalence between 1999 and 2014–15 was concomitant with measurable changes in adolescent social interactions. The UKMenCar4 survey identified reduced participation in carriage-promoting activities, such as smoking, kissing, and attending pubs and nightclubs, compared with UKMenCar1. Changing adolescent behaviour in the UK has been observed in the declining rates of smoking, sexual intercourse, pregnancy, and new sexually transmitted infections reported in national surveillance studies^31^, ^32^ and two large contemporaneous birth cohorts.^33^ There were major social and policy changes over this period, including the introduction of a national smoking ban (2006–07), more rigorous identity checks for admission into pubs and nightclubs, and increased use of mobile telephones and social media, resulting in less direct interpersonal contact. All these influences on adolescent behaviour are likely to affect the transmission of meningococci between hosts, which is a prerequisite to invasive disease. Behavioural changes might, therefore, have effects on the incidence of invasive meningococcal disease, alongside other targeted interventions, including vaccination.

These four UKMenCar surveys were cross-sectional, the most appropriate study design to estimate carriage prevalence in the absence of an intervention or control group. All four UK carriage surveys were done with the same microbiological methods for identification of meningococci, allowing comparisons of results.^8^, ^11^, ^12^, ^13^ The molecular methods to derive nucleotide sequence data for the characterisation of MLST loci and genogroup were technically different between UKMenCar1–3 and UKMenCar4. From retrospective WGS of 498 UKMenCar1 isolates, there were 13 (3%) isolates with MLST discrepancies between PCR-based sequencing and WGS (data not shown). In all four surveys, regional variability was observed among sampling centres (appendix pp 3–4), but these surveys included broadly equivalent populations (appendix pp 6, 8) and consistent study design. Although 2085 (9·6%) of 21 726 swabs could not be cultured and were excluded for technical reasons, overall recruitment excluding these swabs (19 641 participants) exceeded the sample size of 18 000 on which the study was powered. Additionally, 41 putative *Neisseria* spp isolates were excluded as they could not be re-cultured for further serological or genomic analysis, and because we could not confirm their species, they could not contribute to carriage estimates. *N lactamica*, a closely related oropharyngeal commensal organism, has a different demographic pattern of carriage and potential risk factors to *N meningitidis*. It was notable that *N lactamica* carriage prevalence was similar between UKMenCar1 and UKMenCar4; as a result, the ratio of *N meningitidis* to *N lactamica* changed from 17:1 (in 1999) to 6:1 (in 2014–15).

Findings of UKMenCar1–4, which were done in periods of high and low disease incidence in the UK, are consistent with an association between lower incidence of invasive meningococcal disease and lower prevalence of circulating meningococci in adolescents, which in turn is associated with changing social behaviours. Our observations have global implications for modification of public health interventions and vaccine policy to reduce the morbidity and mortality resulting from invasive meningococcal disease and other pathogens residing in the respiratory tract.

**This online publication has been corrected. The corrected version first appeared at thelancet.com on April 22, 2021**

## Data sharing

Short-read sequence data are available from the European Nucleotide Archive (study reference PRJEB14319), with individual European Nucleotide Archive accession run identifiers accessible on PubMLST. All genomes and metadata for the UKMenCar4 study, and all MLST data for UKMenCar1–3 are publicly available through PubMLST.
