# Correction to *Lancet Infect Dis* 2021; 21: 677–87

**DOI:** 10.1016/S1473-3099(21)00229-2

**Published:** 2021-05

*MacLennan JM, Rodrigues CMC, Bratcher HB, et al. Meningococcal carriage in periods of high and low invasive meningococcal disease incidence in the UK: comparison of UKMenCar1–4 cross-sectional survey results.* Lancet Infect Dis *2021; **21:** 677–87—*In this Article, the references 26–33 were incorrectly numbered in the text and in the reference list. This correction has been made to the online version as of April 22, 2021, and the printed version is correct.

