# Correction to *Lancet Infect Dis* 2021; 21: 677–87

**DOI:** 10.1016/S1473-3099(21)00266-8

**Published:** 2021-07

*MacLennan JM, Rodrigues CMC, Bratcher HB, et al. Meningococcal carriage in periods of high and low invasive meningococcal disease incidence in the UK: comparison of UKMenCar1–4 cross-sectional survey results.* Lancet Infect Dis *2021; **21:** 677–87*—In this Article, “18·9%; 122/13 679” in paragraph eight of the Results section should read “8·9%; 1221/13 679”. Additionally, the y-axis for “Disease incidence (cases per 100 000)” on the graphs for Genogroups C, W, and Y in figure 3, and the y-axis for “Measured carriage prevalence (%)” on the graph for Genogroup B in figure 3 were incorrect and have been fixed. These corrections have been made to the online version as of May 6, 2021.

