# Supplementary material for: Meningococcal carriage in periods of high and low invasive meningococcal disease incidence in the UK: comparison of UKMenCar1–4 cross-sectional survey results
Source: Lancet Infect Dis. 2021 May;21(5):677–87. doi: 10.1016/S1473-3099(20)30842-2 (PMC8064914; doi:10.1016/S1473-3099(20)30842-2)
Supplement: Supplementary appendix [file mmc1.pdf]

THE LANCET
Infectious Diseases

Supplementary appendix

This appendix formed part of the original submission and has been peer reviewed. We post it as supplied by the authors.

Supplement to: MacLennan JM, Rodrigues CMC, Bratcher HB, et al. Meningococcal carriage in periods of high and low invasive meningococcal disease incidence in the UK: comparison of UKMenCar1–4 cross-sectional survey results. *Lancet Infect Dis* 2021; published online Jan 19. https://doi.org/10.1016/S1473-3099(20)30842-2.

Supplementary data

(a)

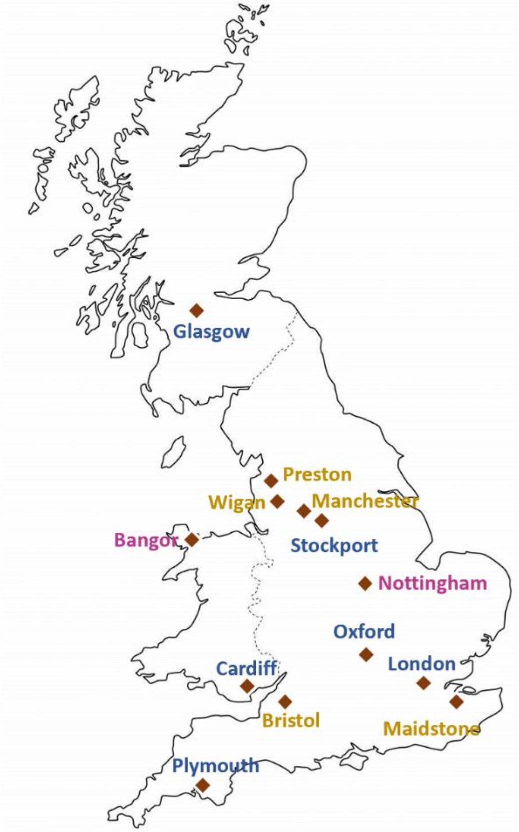

(b)

Sampling centre	UKMenCar1	UKMenCar2	UKMenCar3	UKMenCar4
Plymouth	1,383	2,695	3,470	2,250
Maidstone	-	-	-	548
Bristol	-	-	-	1,817
Cardiff	1,712	1,711	1,640	3,066
London	-	-	-	1,923
Oxford	2,391	2,124	2,433	2,138
Bangor	971	1,086	1,015	-
Nottingham	1,648	2,256	2,549	-
Stockport	2,905	3,435	3,513	2,327
Manchester	-	-	-	1,200
Wigan	-	-	-	774
Preston	-	-	-	1,004
Glasgow	2,891	2,988	2,949	2,626
Total	13,901	16,295	17,569	19,641

Figure 6 Geographical representation of the UKMenCar survey sampling centres (a) For UKMenCar1 (n=13,901), 2 (n=16,295) and 3 (n=17,569) eight centres participated, those coloured pink and blue. For UKMenCar4 (n=19,641) there were eleven sampling centres that took part, those coloured blue and gold. The six centres in blue took part in all four surveys. (b) The number of participants swabs included in the analysis from each sampling centre for the four carriage studies. Cultures from London were not available for analysis for UKMenCar1-3 due to technical issues[9,12].

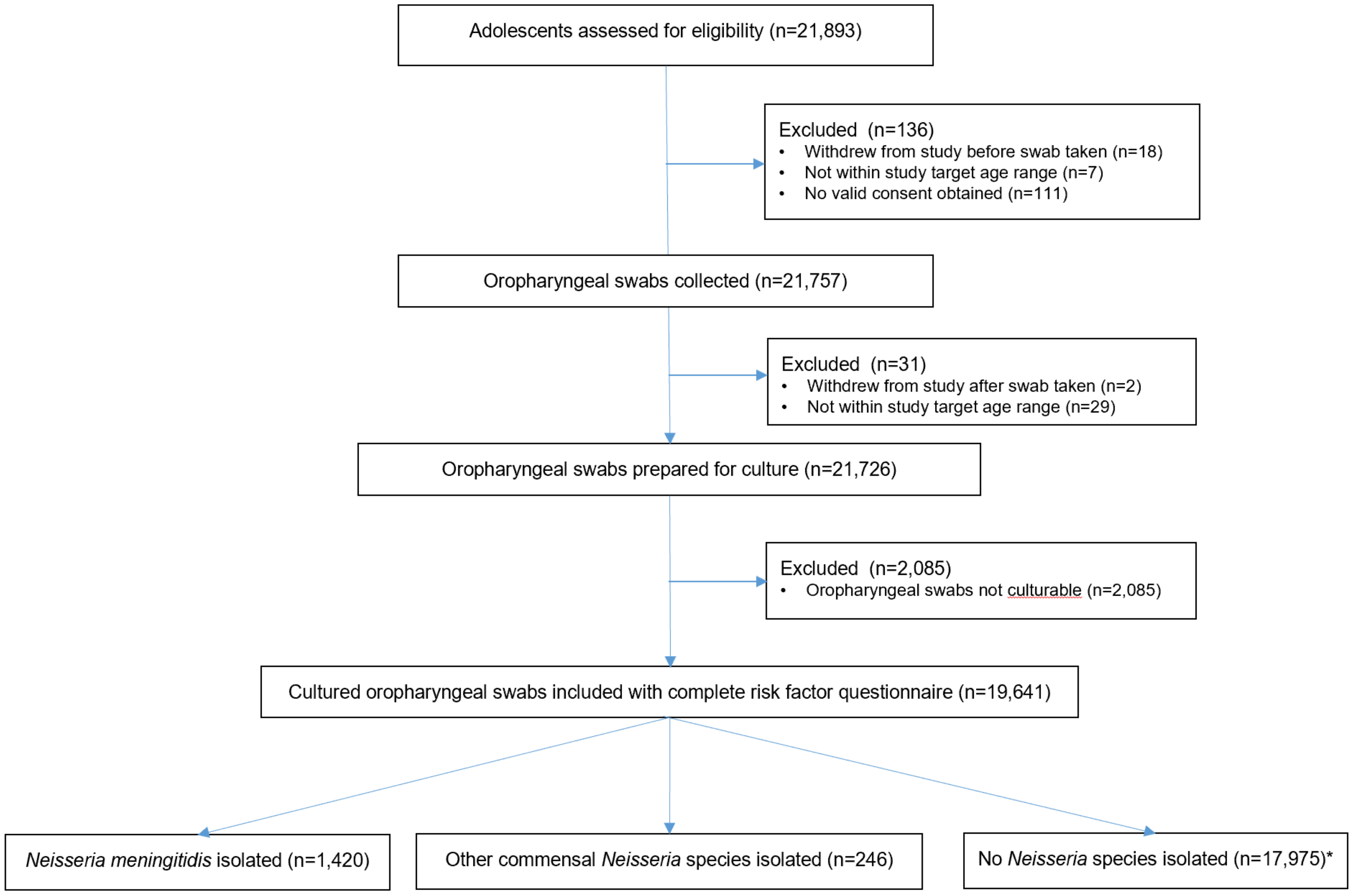

Figure 7 UKMenCar4 survey profile Adolescents aged 15-19 years old were recruited from eleven UK sampling centres, with the aim of recruiting 18,000 participants to adequately power the study for the detection of rare variants. There were 21,893 adolescents assessed for eligibility and 19,641 participants were included in the final analysis of meningococcal carriage and risk factor participation.

Table 2 Carriage prevalence by sampling site for UKMenCar1-4 studies For each study centre (shown geographically south to north of the UK), the number of carriage isolates is shown (n/N) and the carriage point prevalence for each of the centres participating in all four UKMenCar surveys, compared to the overall carriage prevalence. Eight centres took part in the UKMenCar1-3 surveys and eleven centres in UKMenCar4, though isolates from London were not included in the UKMenCar1-3 analysis due to technical issues[9,12]. The number of participants recruited at each sampling centre is in Figure 6 supplementary.

	**UKMenCar1**		**UKMenCar2**		**UKMenCar3**		**UKMenCar4**	
	**n/N**	**%**	**n/N**	**%**	**n/N**	**%**	**n/N**	**%**
**Overall**	2,306/13,901	16·59	2,873/16,295	17·63	3,283/17,569	18·69	1,420/19,641	7·23
**Plymouth**	107/1,383	7·74	395/2,695	14·66	684/3,470	19·71	121/2,250	5·38
**Maidstone**	-	-	-	-	-	-	16/548	2·91
**Bristol**	-	-	-	-	-	-	133/1,817	7·32
**Cardiff**	234/1,712	13·67	259/1,711	15·14	257/1,640	15·67	264/3,066	8·61
**London**	-	-	-	-	-	-	29/1,923	1·51
**Oxford**	526/2,391	22·00	405/2,124	19·07	454/2,433	18·66	149/2,138	6·97
**Bangor**	183/971	18·85	198/1,086	18·23	222/1,015	21·87		
**Nottingham**	285/1,648	17·29	420/2,256	18·62	387/2,549	15·18		
**Stockport**	671/2,905	23·10	736/3,435	21·43	789/3,513	22·46	244/2,327	10·5
**Manchester**	-	-	-	-	-	-	94/1,200	7·83
**Wigan**	-	-	-	-	-	-	101/774	13·1
**Preston**	-	-	-	-	-	-	110/1,004	11·0
**Glasgow**	300/2,891	10·38	460/2,988	15·39	490/2,949	16·62	159/2,626	6·06

Figure 8 Carriage prevalence by sampling site for UKMenCar1-4 studies For each study centre (shown geographically south to north of the UK), the carriage point prevalence is shown with 95% confidence intervals for each of the centres participating in all four UKMenCar surveys, compared to the overall carriage prevalence. Eight centres took part in the UKMenCar1-3 surveys and eleven centres in UKMenCar4, though isolates from London were not included in the UKMenCar1-3 analysis due to technical issues[9,12]. The number of participants recruited at each sampling centre is in Figure 6 supplementary.

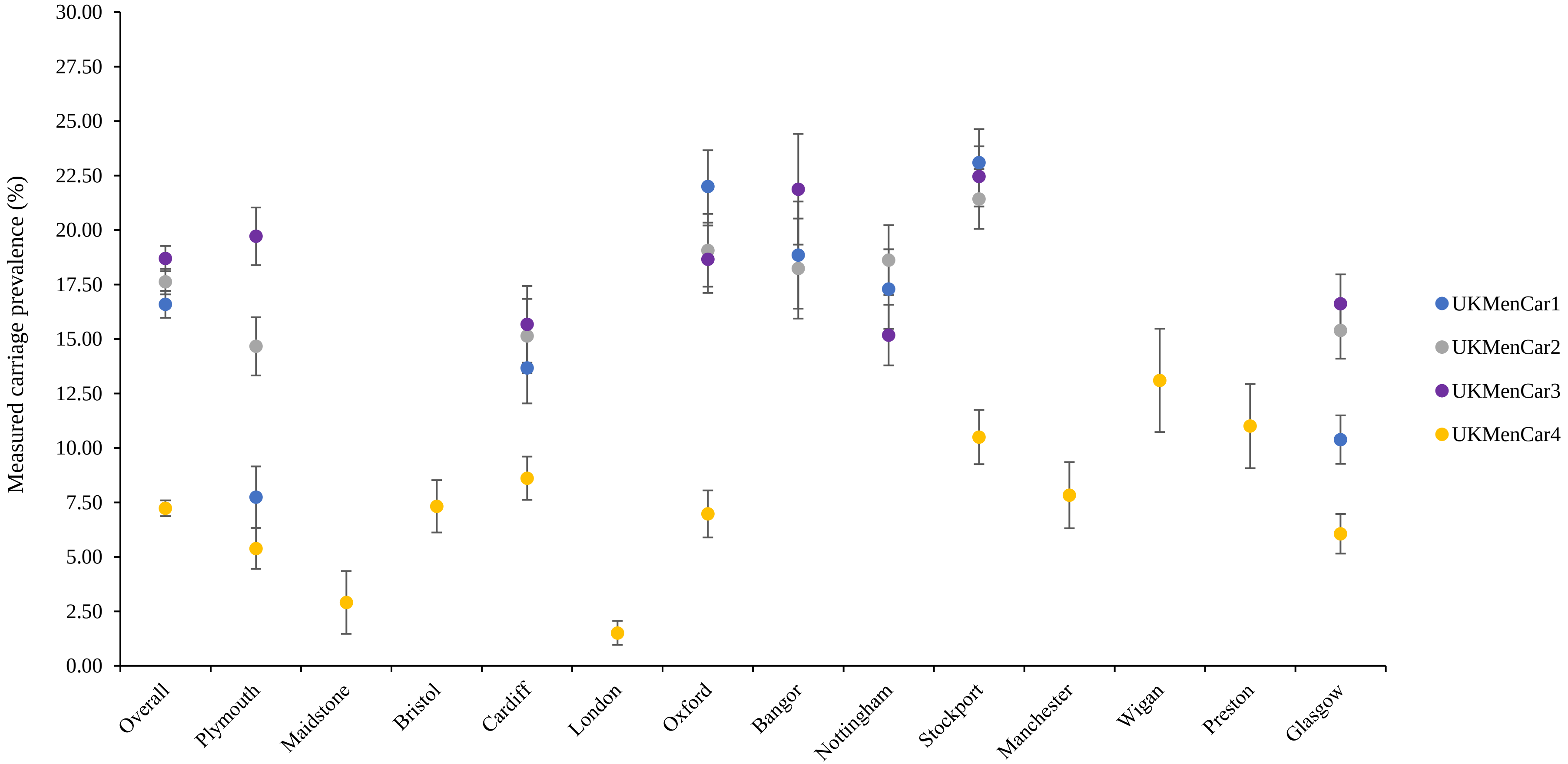

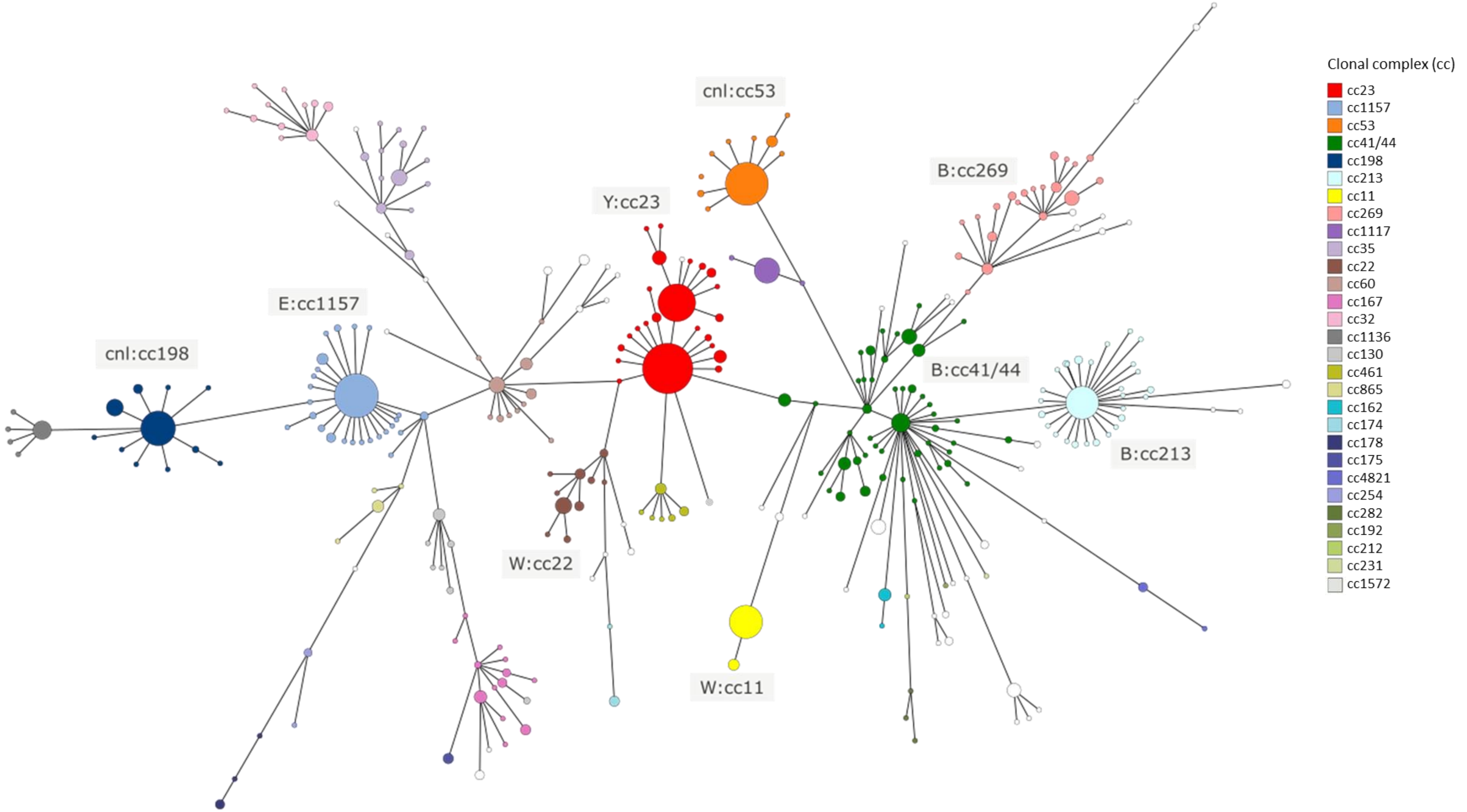

Figure 9 Minimum-spanning tree of carriage *Neisseria meningitidis* genomes from UK adolescents in 2014/15. All 1,420 meningococcal carriage isolates from 2014/15 were analysed using seven locus multilocus sequence typing (MLST) and the relationships among clonal complexes (ccs) are visualised using GrapeTree software. Different ccs are shown by colour, unfilled nodes represent incomplete MLST profiles, and the size of the circles is proportional to the number of isolates. Predominant genogroups are shown in grey boxes with their associated clonal complexes.

Table 3 Demographic information about UKMenCar4 survey participants. Detailed breakdown of participant demographics shown by number of oropharyngeal swabs cultured (n=19,641), those with meningococcal carriage (n=1,420) and those with other *Neisseria* species identified (n=246). Weighted proportion represents the carriage (%) by demographic stratum. UKMenCar4 surveyed 1.74% of the UK birth cohort for 2014 and 2015. The self-reported ethnicity of participants was broadly similar to UK population estimates for ethnic group for 2014-16: White British 78.7%; Asian/Asian British 8.0%; Black/African/Caribbean/Black British 3.5%; Mixed/Multiple ethnic groups 1.8%; Other ethnic group 1.9%.
(https://www.ons.gov.uk/peoplepopulationandcommunity/populationandmigration/populationestimates/articles/researchreportonpopulationestimatesbyethnicgroupandreligion/2019-12-04#population-estimates-by-ethnic-group)

	**Oropharyngeal swabs cultured (n=19,641)**	**Confirmed *Neisseria meningitidis* (n=1,420)**			**Confirmed other *Neisseria* species (n=246)**		
	**No. (%)**	**No. (%)**	**% weighted proportion**	**% carriage prevalence**	**No. (%)**	**% weighted proportion**	**% carriage prevalence**
**Age (years)**							
15	380 (1·93)	15 (1·06)	3·95	0·08	5 (2·03)	1·32	0·03
16	6,287 (32·01)	379 (26·69)	6·03	1·93	82 (33·33)	1·30	0·42
17	8,667 (44·13)	618 (43·52)	7·13	3·15	107 (43·50)	1·23	0·54
18	3,628 (18·47)	345 (24·30)	9·51	1·76	46 (18·70)	1·27	0·23
19	679 (3·46)	63 (4·44)	9·28	0·32	6 (2·44)	0·88	0·03
**Gender**							
Male	8,242 (41·96)	689 (48·52)	8·36	3·51	85 (34·55)	1·03	0·43
Female	11,332 (57·70)	725 (51·06)	6·49	3·69	161 (65·45)	1·42	0·82
Not stated	67 (0·34)	6 (0·42)	8·96	0·03	0 (0·00)	0·00	0·00
**School/College year**							
12	10,624 (54·09)	615 (43·31)	5·79	3·13	130 (52·85)	1·22	0·66
13	7,424 (37·80)	668 (47·04)	9·00	3·40	97 (39·43)	1·31	0·49
Not in formal education	47 (0·24)	8 (0·56)	17·02	0·04	0 (0·00)	0·00	0·00
Other	1,431 (7·29)	118 (8·31)	8·25	0·60	19 (7·72)	1·33	0·10
Not stated	115 (0·59)	11 (0·77)	9·57	0·06	0 (0·00)	0·00	0·00
**Ethnicity**							
White	15,881 (80·86)	1,286 (90·56)	8·10	6·55	197 (80·08)	1·24	1·00
Asian descent	1,877 (9·56)	68 (4·79)	3·62	0·35	26 (10·57)	1·39	0·13
Black decent	875 (4·45)	20 (1·41)	2·29	0·10	9 (3·66)	1·03	0·05
Mixed ethnicity	634 (3·23)	36 (2·54)	5·68	0·18	11 (4·47)	1·74	0·06
Other ethnic group	336 (1·71)	9 (0·63)	2·68	0·05	3 (1·22)	0·89	0·02
Not stated	38 (0·19)	1 (0·07)	2·63	0·01	0 (0·00)	0·00	0·00

Table 4 Odds ratio for carriage by age for UKMenCar1 and UKMenCar4 The odds ratio is calculated with 95% confidence intervals (CI) for each age group in the survey with n=13,901 participants in UKMenCar1 (1999) and n=19,641 participants in UKMenCar4 (2014/15).

Age	OR for carriage 1999	95% CI 1999	OR for carriage 2014/15	95% CI 2014/15
**15**	1		1	
**16**	1·35	1·09-1·67	1·56	0·92-2·64
**17**	1·82	1·47-2·25	1·87	1·11-3·15
**18/19**	2·41	1·90-3·05	2·55	1·50-4·31

Table 5 Demographic information about UKMenCar1-3 survey participants. Detailed breakdown of participant demographics is shown for the UKMenCar1-3 studies performed in 1999, 2000 and 2001 respectively. The number of oropharyngeal swabs cultured (1999 n=13,901, 2000 n=16,295, 2001 n=17,569), those with meningococcal carriage (1999 n=2,306, 2000 n=2,873, 2001 n=3,283). Data regarding ethnicity was not collected for UKMenCar1-3.

	**UKMenCar1 (1999)**			**UKMenCar2 (2000)**			**UKMenCar3 (2001)**		
	**Oropharyngeal swabs cultured (n=13,901)**	**Confirmed *Neisseria meningitidis* (n=2,306)**		**Oropharyngeal swabs cultured (n=16,295)**	**Confirmed *Neisseria meningitidis* (n=2,873)**		**Oropharyngeal swabs cultured (n=17,569)**	**Confirmed *Neisseria meningitidis* (n=3,283)**	
	**No. (%)**	**No. (%)**	**% carriage prevalence**	**No. (%)**	**No. (%)**	**% carriage prevalence**	**No. (%)**	**No. (%)**	**% carriage prevalence**
**Age (years)**									
15	959 (6·90)	105 (4·55)	0·76	582 (3·57)	88 (3·06)	0·54	524 (2·99)	89 (2·71)	0·51
16	5,835 (41·98)	832 (36·08)	5·99	7,416 (45·51)	1,143 (39·78)	7·01	8,138 (46·32)	1,380 (42·03)	7·85
17	5,584 (40·17)	1,020 (44·23)	7·34	6,182 (37·94)	1,191 (41·45)	7·31	7,035 (40·04)	1,367 (41·64)	7·78
18	1,350 (9·71)	301 (13·05)	2·17	1,788 (10·97)	380 (13·23)	2·33	1,682 (9·57)	396 (12·06)	2·25
19	173 (1·24)	48 (2·08)	0·35	327 (2·01)	71 (2·47)	0·44	190 (1·08)	51 (1·55)	0·29
**Gender**									
Male	6,862 (49·36)	1,165 (50·52)	8·38	7,680 (47·13)	1,310 (45·60)	8·04	8,322 (47·37)	1,546 (47·09)	8·80
Female	7,039 (50·64)	1,141 (49·48)	8·21	8,614 (52·86)	1,563 (54·40)	9·59	9,247 (52·63)	1,737 (52·91)	9·89
Not stated	0 (0·0)	0 (0·0)	0 (0·0)	1 (0·01)	0 (0·0)	0 (0·0)	0 (0·0)	0 (0·0)	0 (0·0)
**School/College year**									
12	7,257 (52·20)	1,090 (47·27)	7·84	9,657 (59·26)	1,527 (53·15)	9·37	10,259 (58·39)	1,787 (54·43)	10·17
13	4,856 (34·93)	880 (38·16)	6·33	5,475 (33·60)	1,122 (39·05)	6·89	6,309 (35·91)	1,274 (38·81)	7·25
Other	1,680 (12·09)	308 (13·36)	2·22	1,064 (6·53)	203 (7·07)	1·25	894 (5·09)	204 (6·21)	1·16
Not stated	108 (0·78)	28 (1·21)	0·20	99 (0·61)	21 (0·73)	0·13	107 (0·61)	18 (0·55)	0·10
**Ethnicity**	Data not collected in UKMenCar1-3 survey questionnaires								

Table 6 Measured carriage prevalence for UKMenCar1-4 surveys. The measured carriage prevalence by genogroup and by year for all UKMenCar surveys, shown as proportion and 95% confidence interval (95% CI) of meningococcal carriage (1999 n=2,306, 2000 n=2,873, 2001 n=3,283, 2014/15 n=1,420) from oropharyngeal swabs cultured (1999 n=13,901, 2000 n=16,295, 2001 n=17,569, 2014/15 n=19,641).

	**UKMenCar1 (n=13,901) No. (%, 95% CI)**	**UKMenCar2 (n=16,295) No. (%, 95% CI)**	**UKMenCar3 (n=17,569) No. (%, 95% CI)**	**UKMenCar4 (n=19,641) No. (%, 95% CI)**
**B**	785 (5·65, 5·28-6·04)	994 (6·10, 5·74-6·48)	1,180 (6·72, 6·36-7·10)	346 (1·76, 1·58-1·96)
**C**	139 (1·00, 0·85-1·18)	92 (0·56, 0·46-0·69)	91 (0·52, 0·42-0·64)	11 (0·06, 0·03-0·10)
**W**	242 (1·74, 1·54-1·97)	379 (2·33, 2·11-2·57)	414 (2·36, 2·14-2·59)	99 (0·50, 0·41-0·61)
**Y**	239 (1·72, 1·52-1·95)	300 (1·84, 1·65-2·06)	328 (1·87, 1·68-2·08)	349 (1·78, 1·60-1·97)

Table 7 Relative carriage prevalence for UKMenCar1-4 surveys by clonal complex. The relative carriage prevalence is shown for the nine most frequently occurring clonal complexes of meningococci in UKMenCar4 (2014/15) as proportions of all meningococci isolated per survey. The total meningococci isolated in each carriage survey: 1999 n=2,306, 2000 n=2,873, 2001 n=3,283, 2014/15 n=1,420 from oropharyngeal swabs cultured (1999 n=13,901, 2000 n=16,295, 2001 n=17,569, 2014/15 n=19,641).

	**1999 (n=13,901) No. (%)**	**2000 (n=16,295) No. (%)**	**2001 (n=17,569) No. (%)**	**2014/15 (n=19,641) No. (%)**	**Rate ratio 2014/15:1999 (95% CI)**	**Rate ratio 2014/15:2001 (95% CI)**	**p value for 2014/15:2001**
**Total meningococci**	**2,306 (16·6)**	**2,873 (17·6)**	**3,283 (18·7)**	**1,420 (7·2)**	**0·44 (0·41-0·46)**	**0·39 (0·36-0·41)**	**$<2{\cdot}2\text{x}10^{-16}$**
**Clonal complexes**							
**cc23**	91 (3·95)	106 (3·69)	154 (4·69)	301 (21·20)	5·37 (4·29-6·73)	4·52 (3·76-5·43)	$<2{\cdot}2\text{x}10^{-16}$
**cc1157**	106 (4·60)	152 (5·29)	191 (5·82)	156 (10·99)	2·39 (1·88-3·03)	1·89 (1·54-2·31)	$1{\cdot}4\text{x}10^{-8}$
**cc53**	191 (8·28)	264 (9·19)	342 (10·42)	128 (9·01)	1·09 (0·88-1·35)	0·87 (0·71-1·05)	0·200
**cc41/44**	349 (15·13)	389 (13·54)	425 (12·95)	116 (8·17)	0·54 (0·44-0·66)	0·63 (0·52-0·77)	$2{\cdot}8\text{x}10^{-5}$
**cc213**	153 (6·63)	305 (10·62)	348 (10·60)	101 (7·11)	1·07 (0·84-1·37)	0·67 (0·54-0·83)	0·00077
**cc198**	87 (3·77)	109 (3·79)	148 (4·51)	100 (7·04)	1·87 (1·41-2·47)	1·56 (1·22-2·00)	0·0010
**cc11**	43 (1·86)	26 (0·90)	12 (0·37)	73 (5·14)	2·76 (1·90-4·00)	14·06 (7·66-25·82)	$<2{\cdot}2\text{x}10^{-16}$
**cc269**	88 (3·82)	114 (3·97)	165 (5·03)	52 (3·66)	0·96 (0·69-1·34)	0·73 (0·54-0·99)	0·060
**cc22**	272 (11·80)	407 (14·17)	430 (13·10)	33 (2·32)	0·20 (0·14-0·28)	0·18 (0·13-0·25)	$2{\cdot}2\text{x}10^{-16}$

(a)

Vaccine and carriage study

LABEL

……………………….Health Authority

Please put a distinct **tick** or **cross** in the relevant box
or fill in details clearly.

This information is completely confidential.
It will not be seen by any members of the school staff.

Name of school………………………………Date ___/___/___

Q1 Surname

☐☐☐☐☐☐☐☐☐☐☐☐☐☐☐☐☐☐☐☐

Q2 First name(s)

☐☐☐☐☐☐☐☐☐☐☐☐☐☐☐☐☐☐☐

Q3 I am

male ☐ female ☐

Q4 Please enter your date of birth

Day ☐☐ Month ☐☐ Year ☐☐

Q5 Which school year are you in?

Year 12 Lower sixth ☐ Year 13 Upper sixth ☐ Other ☐

Q6 What is the post code of your home address?

☐☐☐☐ ☐☐☐

Q7 How many people live in your household?

1 ☐ 2 ☐ 3 ☐ 4 ☐ 5 ☐ 6 ☐ 7 or more ☐

Q8 What is the total number of rooms in your house, excluding bathrooms and toilets?

☐☐

Please turn over

Q9 How many people usually sleep in the same bedroom as yourself

1 (myself only) ☐ 2 ☐ 3 or more ☐

Q 10 Have you ever been given a vaccination against meningococcal meningitis in the last 5 years?

Yes ☐ No ☐

Q 11 Are you **currently** taking or recently stopped taking antibiotics?

Yes currently taking ☐ stopped in last week ☐ stopped in last month ☐ not taken in past month ☐

Q 12 How many cigarettes do you smoke in a typical day?

None ☐ 1-5 ☐ 6-10 ☐ 11-20 ☐ 21 or more ☐

Q 13 Does any **other** person in your house smoke cigarettes?

Do not include yourself yes ☐ no ☐

Q 14 How many days in the last week have you visited a pub or night club?

never	1	2	3	4	5	6	7
☐	☐	☐	☐	☐	☐	☐	☐

Q 15 How many people have you kissed in the last week (snogged, tongues, wet, **only**) ? Not just on lips or cheeks.

none	1	2	3	4	5 or more
☐	☐	☐	☐	☐	☐

Thank you for completing the questionnaire.

(b)

UK Meningococcal Carriage Study

LABEL

Today's Date ☐☐ (Day) ☐☐ (month) 20☐☐ (year) Name of School ....

** This information is completely confidential. It will not be seen by school staff or other students. **

Q1 What is your current age in years? 15 ☐ 16 ☐ 17 ☐ 18 ☐ 19 ☐

Q2 I am: male ☐ female ☐

Q3 What is your home postcode? ☐☐☐☐ ☐☐☐

Q4 Which school year are you in? Year 12 Lower Sixth ☐ Year 13 Upper Sixth ☐ Other e.g. part time college ☐ Not in full time education ☐

Q5 Do you currently have a cold or sore throat? NO ☐ YES ☐

Q6 Are you currently taking or have you recently stopped taking antibiotics?
not taken in the past month ☐ stopped in the last month ☐ stopped in the last week ☐ yes, currently taking ☐

Q7 How many cigarettes do you smoke in a typical day?
0 ☐ 1-5 ☐ 6-10 ☐ 11-20 ☐ more than 20 ☐

Q8 How many times have you smoked an e-cigarette in the last week?
0 ☐ 1-2 ☐ 3-6 ☐ 7 or more ☐

Q9 How many times have you smoked a waterpipe (shisha, hookah, hubbly bubbly) in the last month?
0 ☐ 1 ☐ 2 ☐ 3-4 ☐ 5 or more ☐

Q10 Does any other person at home smoke cigarettes? NO ☐ YES, outside the house ☐ YES, inside the house ☐

Q11 How many days in the last week have you been to a party, pub, bar or night club?
0 ☐ 1 ☐ 2-3 ☐ 4-5 ☐ 6-7 ☐

Q12 How many people have you kissed (kissing with tongues, not just lips or cheeks) in the last week?
0 ☐ 1 ☐ 2-3 ☐ 4 or more ☐

Q13a Do you have a regular girlfriend or boyfriend? NO ☐ YES ☐

Q13b If YES: do they smoke cigarettes? NO ☐ YES ☐
do they smoke shisha? NO ☐ YES ☐

Q14 What is your ethnic group?
White ☐ Asian/Asian British ☐ Black/African/Caribbean/Black British ☐
Mixed/multiple ethnic ☐ Other ethnic group ☐

Thank you for completing this questionnaire

UKMENCAR4 Questionnaire v1.2 11/08/14; REC REF 14/SC/1163

Figure 10 Risk factor questionnaires. The questionnaires were completed by participants of: (a) UKMenCar1-3 studies (1999, 2000, 2001) and (b) UKMenCar4 (2014/15) carriage surveys.
